# A DEVELOPMENTAL–CONTEXTUAL MODEL OF COUPLE SYNCHRONY: CENTRAL TENETS AND EMPIRICAL APPLICATION

**DOI:** 10.1093/geroni/igad104.1593

**Published:** 2023-12-21

**Authors:** Theresa Pauly, Denis Gerstorf, Hans-Werner Wahl, Janina Lüscher, Urte Scholz, Christiane Hoppmann

**Affiliations:** Simon Fraser University, Vancouver, British Columbia, Canada; Humboldt University of Berlin, Berlin, Berlin, Germany; Heidelberg University, Heidelberg, Baden-Wurttemberg, Germany; Swiss Paraplegic Research, Nottwil, Luzern, Switzerland; University of Zurich, Zurich, Zurich, Switzerland; University of British Columbia, Vancouver, British Columbia, Canada

## Abstract

Most current models of social dynamics and health focus on individuals; our developmental-contextual model of couple synchrony (CoSynch) proposes a uniquely dyadic approach and deliberately moves to a N + 1 perspective. CoSynch emphasizes the importance of between-person synchrony for shaping development and health, which refers to the way in which physiological states and health behaviors fluctuate in sync between romantic partners. Synchrony is proposed to follow a u-shaped curve across adulthood, with younger and older couples showing greater synchrony than middle-aged couples, and with greater diversification of synchrony in very old age. We assume that synchrony is shaped by the closeness and shared contexts of couples, and is correlated with individual and dyad characteristics (e.g., attachment). Prior research has linked synchrony to perspective taking and effective collaboration. Thus, synchrony is assumed to be important for supportive interactions in couples managing chronic disease. As an empirical illustration, we apply the CoSynch model to analyze 992 daily life assessments from 11 Swiss couples in which one partner had Type II diabetes. Specifically, we used a smartphone and smartwatch system (DyMand) that captured daily social contexts and heart rate synchrony. Couples showed small to moderate interconnections in their heart rate fluctuations, which tended to be stronger when engaged in conversation with the partner. We will discuss the utility of using wearables to better understand health-relevant social developmental dynamics. Insights from collecting such data could be used to inform future interventions using technology to promote healthy aging and adaptive disease management.

